# Effects of torso mesh density and electrode distribution on the accuracy of electrocardiographic imaging during atrial fibrillation

**DOI:** 10.3389/fphys.2022.908364

**Published:** 2022-08-29

**Authors:** Rubén Molero, Ana González-Ascaso, Ismael Hernández-Romero, David Lundback-Mompó, Andreu M. Climent, María S. Guillem

**Affiliations:** ^1^ ITACA Institute, Universitat Politècnica de València, València, Spain; ^2^ Corify Care SL, Madrid, Spain

**Keywords:** electrocardiographic imaging, geometry, torso, atrial fibrillation, mesh density

## Abstract

**Introduction:** Electrocardiographic Imaging (ECGI) allows computing the electrical activity in the heart non-invasively using geometrical information of the patient and multiple body surface signals. In the present study we investigate the influence of the number of nodes of geometrical meshes and recording ECG electrodes distribution to compute ECGI during atrial fibrillation (AF).

**Methods:** Torso meshes from 100 to 2000 nodes heterogeneously and homogeneously distributed were compared. Signals from nine AF realistic mathematical simulations were used for computing the ECGI. Results for each torso mesh were compared with the ECGI computed with a 4,000 nodes reference torso. In addition, real AF recordings from 25 AF patients were used to compute ECGI in torso meshes from 100 to 1,000 nodes. Results were compared with a reference torso of 2000 nodes. Torsos were remeshed either by reducing the number of nodes while maximizing the overall shape preservation and then assigning the location of the electrodes as the closest node in the new mesh or by forcing the remesher to place a node at each electrode location. Correlation coefficients, relative difference measurements and relative difference of dominant frequencies were computed to evaluate the impact on signal morphology of each torso mesh.

**Results:** For remeshed torsos where electrodes match with a geometrical node in the mesh, all mesh densities presented similar results. On the other hand, in torsos with electrodes assigned to closest nodes in remeshed geometries performance metrics were dependent on mesh densities, with correlation coefficients ranging from 0.53 ± 0.06 to 0.92 ± 0.04 in simulations or from 0.42 ± 0.38 to 0.89 ± 0.2 in patients. Dominant frequency relative errors showed the same trend with values from 1.14 ± 0.26 to 0.55 ± 0.21 Hz in simulations and from 0.91 ± 0.56 to 0.45 ± 0.41 Hz in patients.

**Conclusion:** The effect of mesh density in ECGI is minimal when the location of the electrode is preserved as a node in the mesh. Torso meshes constructed without imposing electrodes to constitute nodes in the torso geometry should contain at least 400 nodes homogeneously distributed so that a distance between nodes is below 4 cm.

## 1 Introduction

Electrocardiographic imaging (ECGI) is a non-invasive technique that can be used to estimate the electrical activity of the heart from surface electrocardiographic signals. ECGI offers multiple clinical applications, such as ablation guidance in atrial fibrillation (AF) patients. ECGI requires to use torso and heart geometries together with electrical recordings from the patient. Firstly, surface electrodes placed over the torso are used to record electrical signals. Additionally, the heart geometry is usually obtained from medical images (magnetic resonance imaging or axial computerized tomography) (Salinet et al., 2021), and the torso geometry can be derived from photogrammetry (Rodrigo et al., 2018), with latter reconstruction creating triangular or polygonal meshes (van der Graaf et al., 2016). Once these elements are acquired, the inverse problem can be solved and epicardial potentials are estimated, which can be used to compute dominant frequencies or rotor-related metrics (Rodrigo et al., 2017a).

The properties of the 3D torso geometry have been proven to affect the calculation of the ECGI. Accurate reconstructions (Messinger-Rapport and Rudy, 1990) of the anatomy of the patient’s body and the use of real dimensions in the torso model (Jamison et al., 2011) show more precise results. Incorporation of inner organs into the geometry of the problem has not shown a major impact on the shape of ECGI potentials (Ramanathan and Rudy, 2001). However, additional geometrical effects should be carefully considered in order to achieve a sufficient resolution.

The objective of this study is to evaluate the repercussion of the number of nodes of the torso geometry mesh and their distribution on the resolution of the ECGI using both AF simulations and real recordings from AF patients. We hypothesized that there is an effect on the ECGI reconstruction quality related to the number of nodes on the torso mesh used independently of the number of ECG electrodes that record the signal. A careful analysis will allow us to establish a threshold to ensure good performance while keeping the computing time as low as possible. We studied two different remeshing situations based on the positioning of body surface electrodes. The first was maintaining the electrodes in the original position while remeshing the rest of the torso to quantify the effect of mesh density and distribution on the morphology of ECGI signals, and our second remeshing alternative was to remesh the whole torso surface to maximize resemblance between original and remeshed volumes, and then we reassign the electrode nodes as those with the smallest Euclidean distance between the original and remeshed torso geometries, in order to quantify the effect of electrode displacement as a consequence of remeshing. We compared the electrocardiographic signals (ECGI) using time metrics: the Pearson’s correlation coefficient (CC), the relative difference measurement (RDM*) and errors in dominant frequency estimation. To obtain the ECGI potentials, we used real torso geometries from AF patients with different geometrical resolutions, 9 electrophysiological AF simulations, and 25 ECGI recordings from AF patients.

## 2 Materials and methods

To analyze the effect related with node variations of torso geometry on the ECGI, we first created the torso models with different numbers and distribution of nodes, then computed the respective inverse electrograms, and finally compared the results using time metrics (CC and RDM*) and dominant frequencies related maps and metrics.

### 2.1 Study population—Data acquisition

#### 2.1.1 Simulation data

Cardiac electrophysiological simulations lasting for 10 s included in this study were created using the same cardiac geometry and different AF episodes. A realistic 3D model of the atrial anatomy composed of 284,578 nodes and 1,353,783 tetrahedrons was used for creating the simulations (Rodrigo et al., 2017b). Variation of ionic current parameters was introduced in I_k,ACH_, I_K1_, I_Na,_ and ICaL to simulate electrical remodeling and allow the maintenance of atrial fibrillation. Fibrotic tissue was modeled by disconnecting a percentage of nodes between 20% and 60% and scar tissue by disconnecting 100% of nodes in the scar region. The system of differential equations was solved by using Runge–Kutta integration on a graphic processors unit (NVIDIA Tesla C2075 6G), (Rodrigo et al., 2017b). AF was induced by implementing an S1 S2 protocol, with the S2 stimulus applied at different locations in the atria, thus producing different AF patterns.

#### 2.1.2 Patient data

The electrical recordings from 25 atrial fibrillation patients from Hospital Gregorio Marañón, Madrid, Spain (Ethics Committee Approval 475/14) described elsewhere (Rodrigo et al., 2020; Molero et al., 2021) were used. To record the signals 57 electrodes distributed on the torso of the patients were employed. The atrial geometries were also obtained from the same patients using Magnetic Resonance Imaging, and the 3D models were segmented through ITK-Snap (Yushkevich et al., 2006) and Autodesk Meshmixer (Schmidt and Singh, 2010). Furthermore, the torso models were obtained from photogrammetry, and 3D geometries consisting of triangular meshes were constructed (Remondino, 2004) and refined with Autodesk Meshmixer.

### 2.2 Data processing

#### 2.2.1 Torso remeshing

**FIGURE 1:**
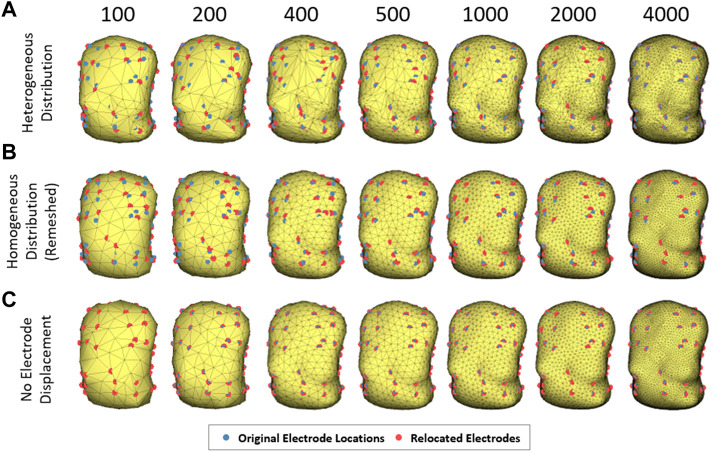
Example of torso models with different number of nodes and node distribution. The electrodes relocated appear in blue and the original locations in red. **(A)** illustrates torsos with irregular mesh distribution, **(B)** with homogeneous distribution and **(C)** torsos maintaining the electrodes in the original position.

**FIGURE 2:**
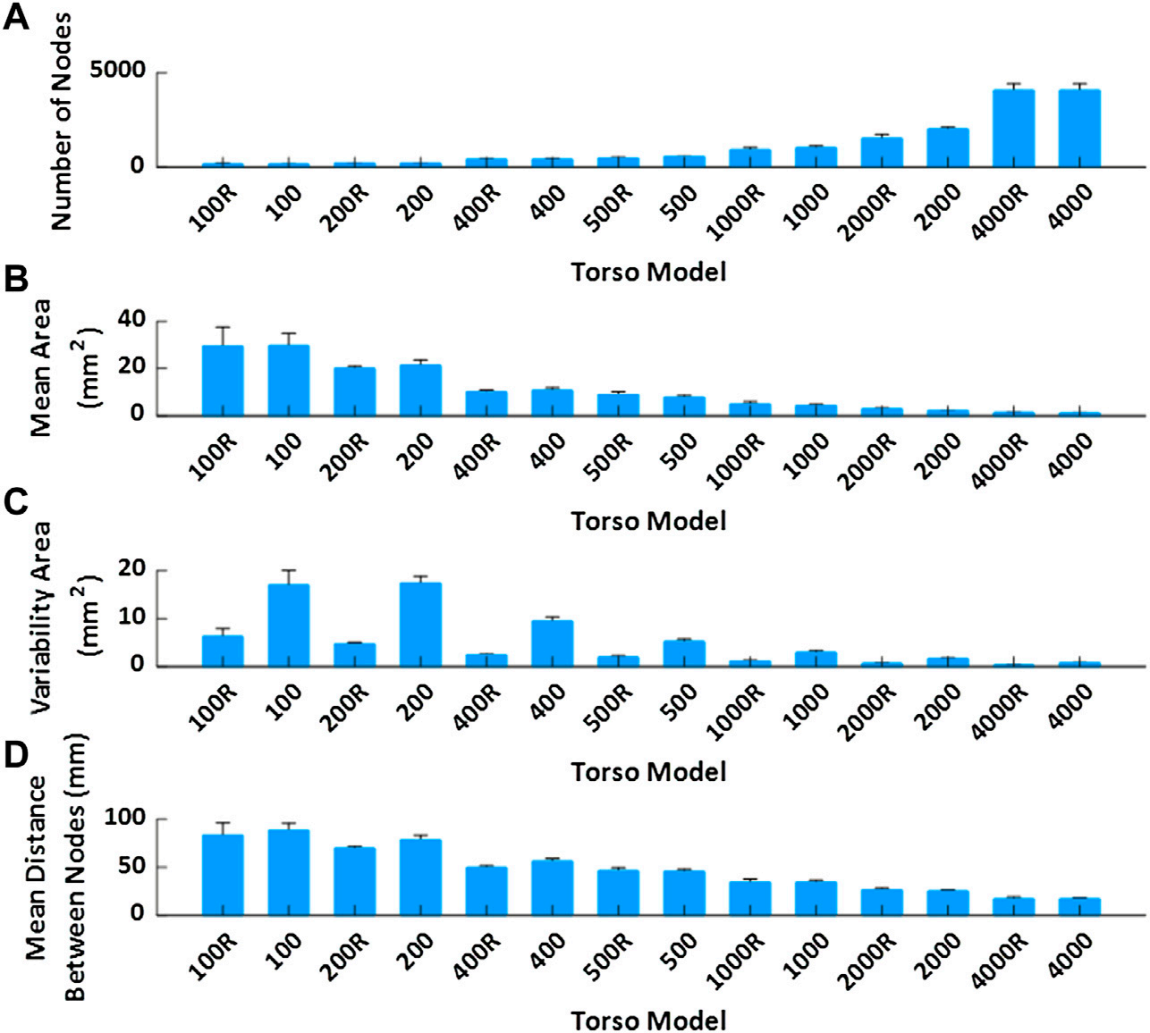
Mean value and standard deviation of torso model properties of the geometries used in the simulation study represented. **(A)**. Mean number of nodes depending on the model. **(B)**. Mean area of the faces. **(C)**. Variability of the area of the faces. **(D)**. Mean distance between nodes of the same triangle.

**FIGURE 3:**
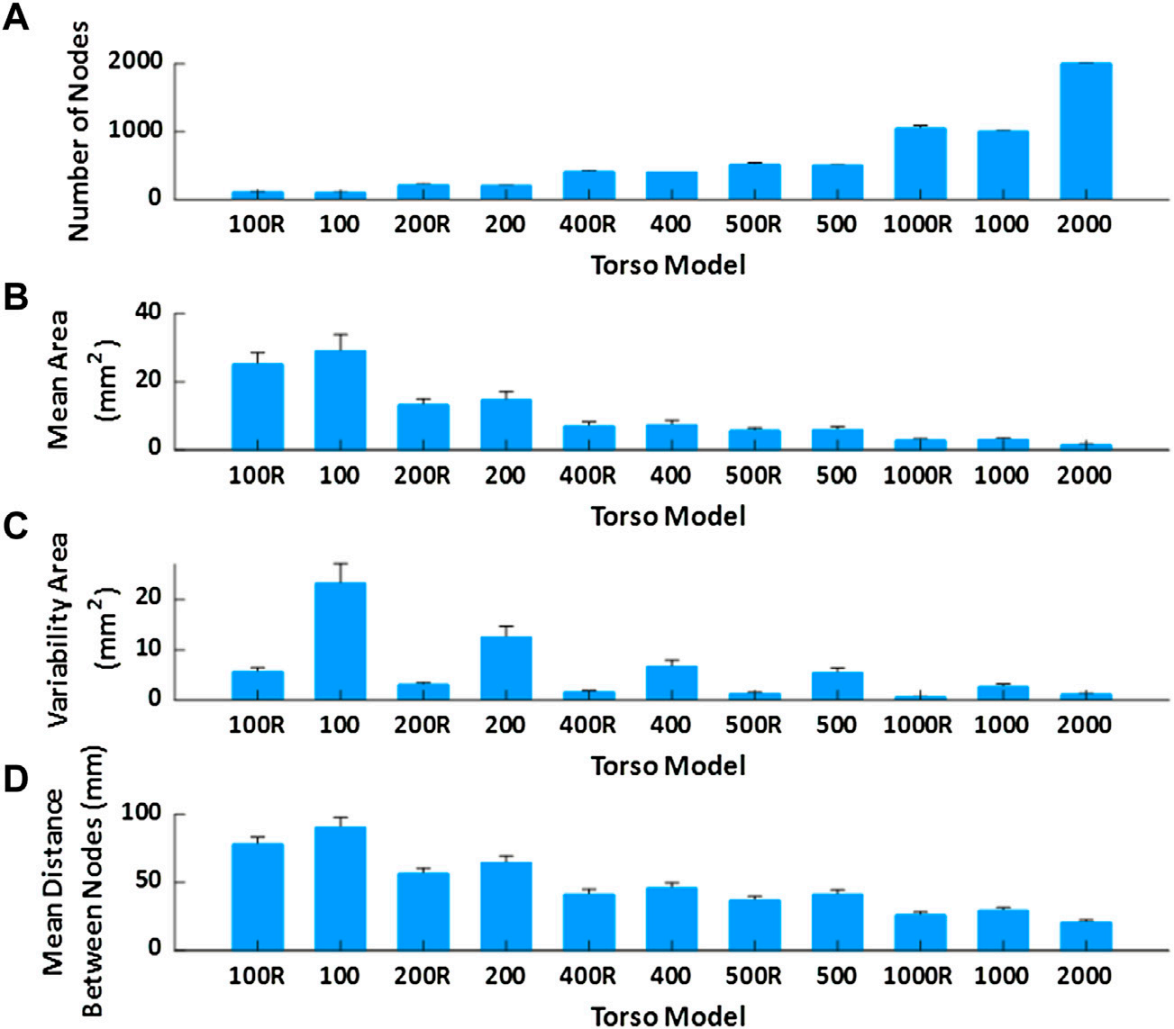
Mean value and standard deviation of torso model properties of the geometries used in the real patient’s study. **(A)**. Mean number of nodes depending on the model. **(B)**. Mean area of the faces. **(C)**. Variability of the area of the faces. **(D)**. Mean distance between nodes of the same triangle.

In order to evaluate the effect of torso mesh density on the morphology of the electrograms after resolution of the inverse problem of electrocardiography, we constructed torso meshes with a reduced number of nodes departing from the finest torso meshes available. We used as reference the torso meshes constructed for each patient, constituted of at least 2000 nodes. The epicardial potentials computed for each of the electrophysiological models were placed in the same position as the original heart inside the thorax. In order to calculate body surface potentials for the computer model simulations, we chose 10 different patient meshes of 4,000 nodes. An inhomogeneous remeshing of torso geometries down to 100, 200, 400, 500, and 1,000 nodes for patients (plus a 2000 nodes mesh for cardiac simulations), maximizing shape preservation was performed with MATLAB built-in functions (see Figure 1A). In order to quantify the impact on ECGI resolution of the homogeneity of the distance of the nodes in the mesh, we also constructed meshes with a homogeneous distribution of nodes based on an iterative approach (Manu, 2022) (see Figure 1B). Properties of the different torso meshes used with simulations and patients are displayed in Figures 2, 3, respectively.

For solving the inverse problem of electrocardiography, electrodes have to be located in the torso mesh. We chose the node with the smallest Euclidean distance from each electrode to relocate electrodes on the mesh. In order to evaluate separately the effect of mesh density and electrode relocation, we also constructed downsampled meshes without electrode relocation. For imposing the electrode position in all the meshes, the closest face of the geometry to each electrode was triangulated again, and three new triangles were included joined by the original electrode position (see Figure 1C).

#### 2.2.2 Processing of surface potentials and Electrocardiographic Imaging calculation

In mathematical models, the forward problem of the simulated electrograms was calculated using the boundary element method (BEM) (Pedrón-Torrecilla et al., 2016). Noise was added to the computed surface potentials to obtain a 20 dB signal to noise ratio emulating the noise present in real recordings. The baseline was subtracted, and a low pass filter of 40 Hz was applied. The electrical information related to the nodes representing the 57 electrodes was selected, and the inverse problem was calculated through the BEM, using zero-order Tikhonov regularization and L-curve optimization (Pedrón-Torrecilla et al., 2016).

Body surface signals obtained from each patient with surface electrodes were pre-processed by selecting 5 s and removing the baseline. A 10th order Butterworth was used to band-pass filter between 2 and 45 Hz to eliminate the noise. The Principal Component Analysis (PCA) approach was performed electrode by electrode to cancel the ventricular activity (QRST segment), (Castells et al., 2005).

Once the recorded or simulated body surface signals were processed, the inverse computed electrograms were calculated through BEM using zero-order Tikhonov regularization and L-curve optimization (Pedrón-Torrecilla et al., 2016).

### 2.3 Quality of mesh evaluation metrics

To evaluate the effect of the mesh in the reconstruction of ECGI potentials, the similarity between ECGI signals obtained with finest and sparser torso meshes was evaluated.

Specifically, we used Pearson’s correlation coefficient (CC) and the relative difference measurement (RDM*) (Meijs et al., 1989; Figuera et al., 2016). For both metrics, the temporal version was used (for each node, the CC and RDM* were computed using all the time instants, and the mean and standard deviation across nodes are then calculated).

$${\text{RDM}}^{\text{*}}=\;\sqrt{{\sum_{\text{k}}\left(\frac{{\text{x}}_{\text{k}}}{{\text{‖x}}^{2}\|}-\frac{{\hat{\text{x}}}_{\text{k}}}{{\|\hat{\text{x}}}^{2}\|}\right)}^{2}}$$

### 2.4 Frequency metrics

The dominant frequency (DF) of each node of the cardiac geometry was estimated after the calculation of ECGI using Welch periodogram (2-s Hamming window with a 25% overlap) (Rodrigo et al., 2017a). The absolute difference in DF for each atrial node between the reference and the other models was calculated for both AF simulations and AF patient studies (Figuera et al., 2016).

## 3 Results

### 3.1 Impact of mesh density on Electrocardiographic Imaging reconstruction

**FIGURE 4:**
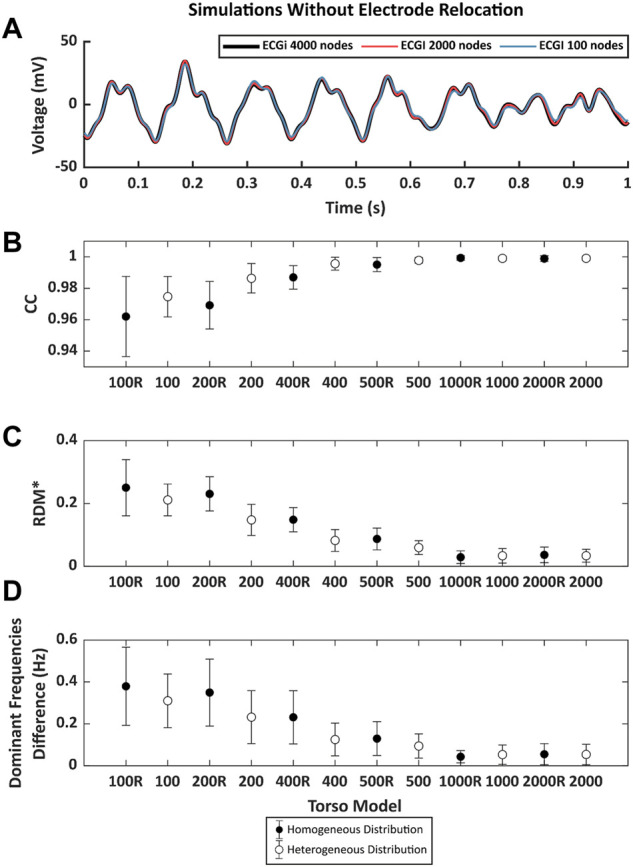
**(A)**. Example of 1 s of inverse computed electrograms obtained with different torso models and the same simulation signals for torsos without electrode relocation. Signal in black was obtained with the reference 4000-node torso, red and blue signals correspond to the ones obtained with torsos of 2000 and 100 nodes respectively. **(B)**. Pearson’s correlation coefficient (CC), **(C)**. relative measurement (RDM*) and **(D)**. mean absolute difference between the reference dominant frequencies (DF) between torso models and from 100 to 2000 nodes. Points in black represent the mean value of the metrics torsos in which the distribution of the nodes is homogeneous and white points represent the torso with nodes heterogeneously placed. Whiskers represent the standard deviation.

Mesh density alone -without electrode relocation-had a limited impact on ECGI signals. In Figure 4A, reconstructed signals with different mesh densities for a sample epicardial node show only subtle differences. Average correlation coefficients remain above 0.96 even for torso meshes with just 100 nodes, and relative errors are below 0.3%, with the lowest CC values of 0.93, Figures 4B,C. The effect of the torso’s node density in the dominant frequencies is depicted in Figure 4D. The observed absolute error decreases with the number of nodes of the mesh, and errors are stabilized below 0.2 Hz with torso meshes with at least 400 nodes.

**FIGURE 5:**
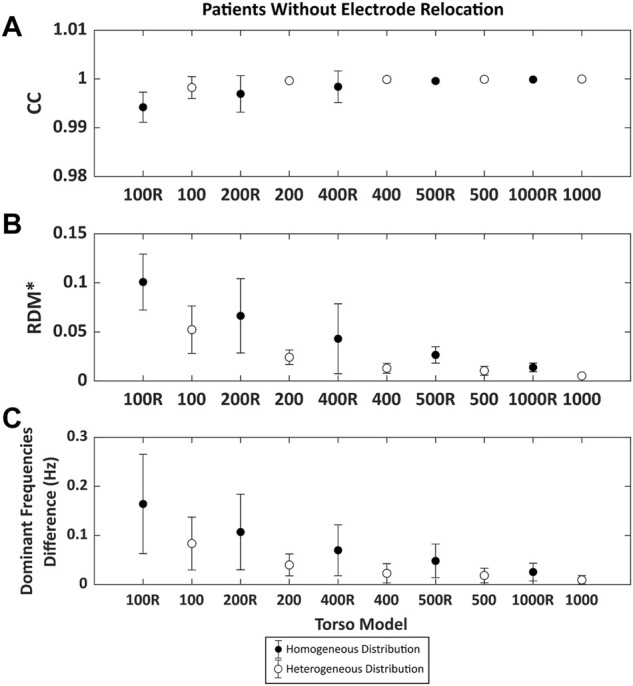
Time metrics obtained comparing the inverse computed electrograms 25 atrial fibrillation patients of the reference and the signals obtained with different torso models without electrode relocation. Points in black represent the torsos in which the distribution of the nodes is homogeneous and white points represent the torso with nodes heterogeneously placed. **(A)**. Pearson’s correlation coefficient (CC) and **(B)**. Relative difference measurement (RDM*). **(C)**. Mean absolute difference between the reference dominant frequencies (DF) between torso models and from 100 to 1,000 nodes.

The same analysis on real AF patient data is shown in Figure 5. Again, CCs were above 0.99 even for meshes with 100 nodes, relative errors were below 0.1 and errors in DF were below 0.2 Hz. CC values for low-density meshes presented very high values, even higher than those obtained for the simulated data. This was because when solving the inverse problem in patients, the optimal regularization parameter was higher than in the simulated cases (∼10^−5^ vs. ∼10^−8^), likely because of the presence of spatial uncertainties in ECGI reconstruction and the presence of different sources of noise on the recorded signals. These larger values of regularization parameters in patients result in smoother ECGI solutions that make the ECGI signal estimation less dependent on mesh resolution.

In addition to the effect of the number of nodes, the type of remeshing affected the quality of the ECGI signal. Results showed that homogenous meshes present lower values of CC and higher values of RDM* and DF errors compared to the heterogeneous distribution of the mesh, which could be attributed to a poorer shape preservation in the homogeneous meshes.

### 3.2 Impact of electrode relocation in low-density torso meshes on Electrocardiographic Imaging reconstruction

**FIGURE 6:**
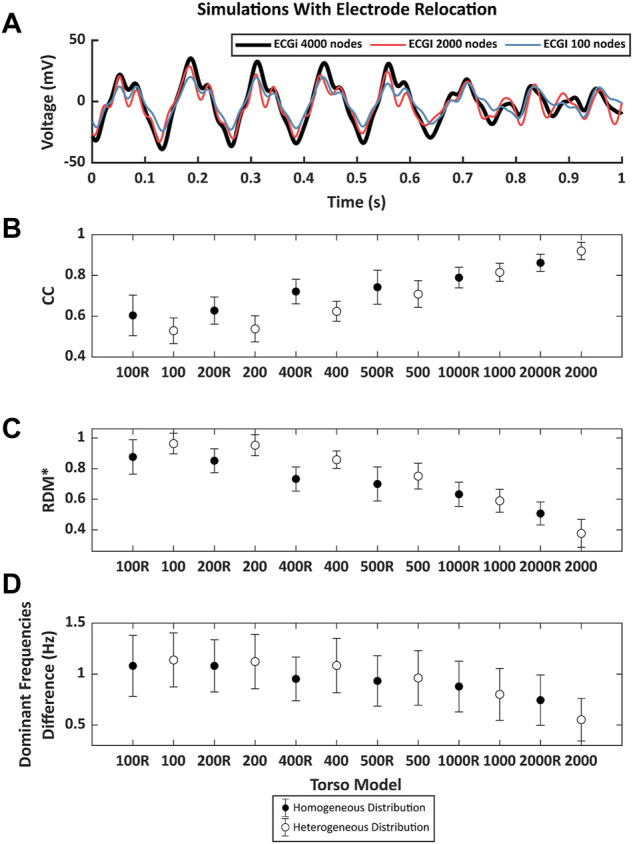
**(A)**. Example of 1 s of inverse computed electrograms obtained with different torso models and the same simulation signals for torsos with the node of the electrode displaced by the remeshing. Signal in black was obtained with the reference 4000-node torso, red and blue signals correspond to the ones obtained with torsos of 2000 and 100 nodes respectively. **(B)**. Pearson’s correlation coefficient (CC), **(C)**. relative measurement (RDM*) and **(D)**. mean absolute difference between the reference DF between torso models and from 100 to 2000 nodes. Points in black represent the mean value of the metrics torsos in which the distribution of the nodes is homogeneous and white points represent the torso with nodes heterogeneously placed. Whiskers represent the standard deviation.

ECGI signals obtained from cardiac electrophysiological simulations and using different torso meshes where the electrode position was relocated to match a mesh node after remeshing present noticeable differences with the reference ECGI signals with the finest torso meshes without electrode relocation (Figure 6). First, an example of simulated ECGI signals of the reference torso with coarser meshes is presented in panel Figure 6A. Although the overall shape of the inversely computed electrograms is preserved for lower mesh densities, some impact of shape morphology can be observed, especially for the sparser meshes (blue line). A global comparison between the signals measured through time-metrics is represented in Figures 6B,C for all the models. The CC and the RDM* show a strong dependency on torso mesh density. A progressive increase is shown for the CC as the number of nodes increases, from 0.53 ± 0.06 for the 100 mesh to 0.92 ± 0.04 for the 2000 node mesh. Besides, the RDM* decreases when the torso is composed with a higher number of vertices from 0.96 ± 0.07 for the 100 mesh down to 0.38 ± 0.09 for the 2000 node mesh. Regular meshes do show better correlation coefficients and RDM* values than irregular meshes with a similar number of nodes, especially for the meshes with a lower number of nodes. For the finer meshes and, therefore, smaller areas of the geometrical faces, slightly better results are observed for the irregular meshes.

Figure 6D shows the differences in DF between the ECGI signals calculated with the torso meshes with 4,000 nodes homogeneously distributed and the remaining models. The largest difference can be observed for the torso with 100 nodes (1.14 ± 0.26 Hz), and it decreases as the number of nodes increases. Differences in the frequencies show higher values when a homogeneous distribution of the electrodes is presented for models with fewer than 1,000 nodes. However, when the number of nodes was 1,000 or higher, these differences were higher in the case of the homogeneous models.

**FIGURE 7:**
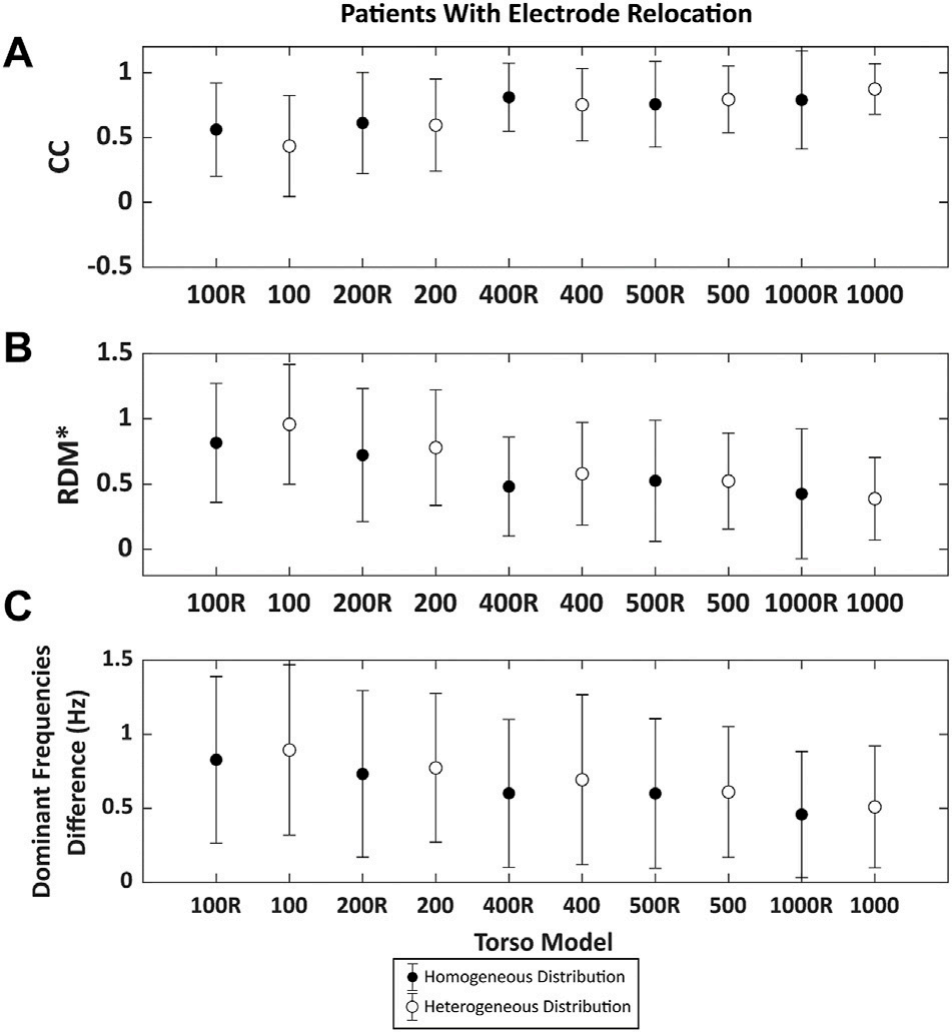
Time metrics obtained comparing the inverse computed electrograms 25 atrial fibrillation patients of the reference and the signals obtained with different torso models with the node of the electrode displaced by the remeshing. Points in black represent the torsos in which the distribution of the nodes is homogeneous and white points represent the torso with nodes heterogeneously placed. **(A)**. Pearson’s correlation coefficient (CC) and **(B)**. Relative difference measurement (RDM*). **(C)**. Mean absolute difference between the reference DF between torso models and from 100 to 1,000 nodes.

The results of the CC and RDM* of the ECGI computed with each torso mesh from real AF patient data are presented in Figure 7. As observed with the computer simulations, the CC values increased, and the RDM* decreased with the number of nodes. Even though the trend is the same as presented in Figure 6, differences are more prominent using real AF signals from patients as compared to simulation data. The correlation coefficient ranged from 0.42 ± 0.38 using the 100 nodes torso and up to 0.87 ± 0.2 with the 1,000 mesh. The RDM* decreases from 0.98 ± 0.45 (100 nodes) to 0.40 ± 0.33 (1000R). Although the results show a more marked effect of the remeshing in real AF signals, both CCs and RDM* values showed a stabilization for torsos above 400 nodes, as in Figure 6.

**FIGURE 8:**
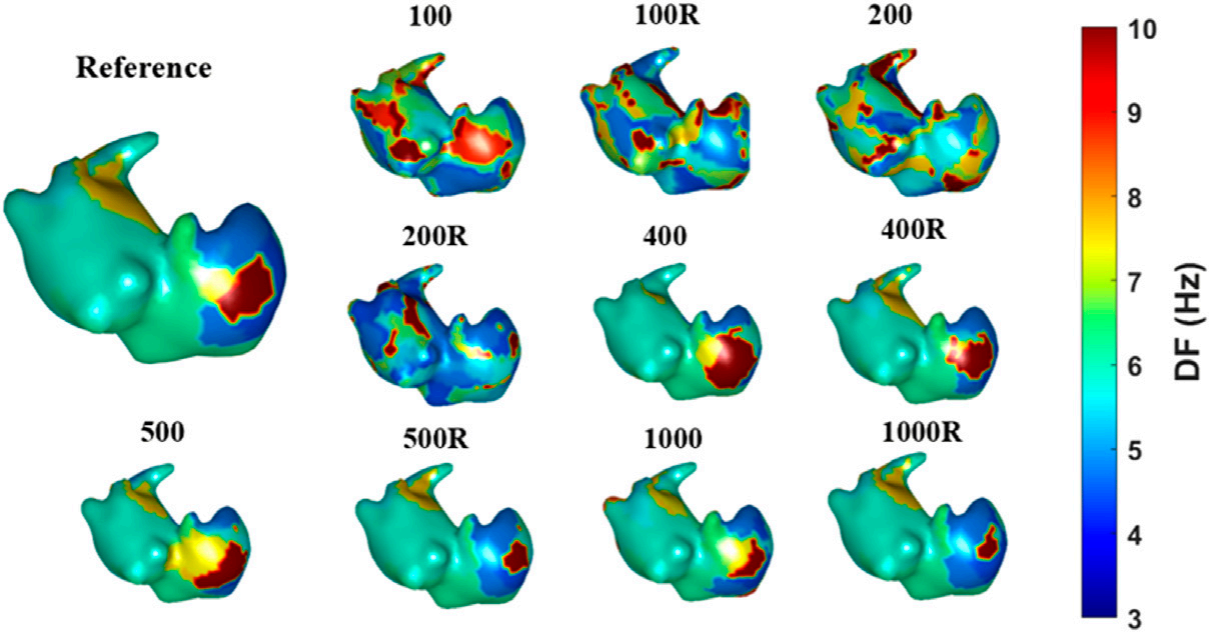
Dominant frequency maps obtained with different torso models for one real patient case.

The calculation of differences in dominant frequencies is shown in Figure 7C. The results presented the same trend as the findings for simulations, and the difference in DF decreases with increasing number of nodes. The largest difference is found for the torso with 100 nodes (0.91 ± 0.56 Hz), while the lowest difference is obtained using the 1000-node torso (0.45 ± 0.41 Hz). In this case we could observe that differences in DFs were lower for the homogeneous torso meshes than their inhomogenous counterparts. DF maps for a sample patient are shown in Figure 8. As the number of vertices increased, the maps looked more similar to the one obtained with the reference torso mesh (2000 nodes). Torso meshes constituted by 400 nodes or less didn’t allow to determine the site with the highest DF, present in the right atrium. In addition, in torso meshes with nodes from 400 to 1,000, the location and extension of the highest dominant frequency area are more similar to the reference ECGI 2000-nodes torso.

## 4 Discussion

In this study, we explored the effect of torso mesh density and homogeneity on ECGI signals for atrial fibrillation simulations and real signals. Firstly, we studied the effect of the number of nodes on torso meshes with imposed nodes matching with the location of the electrodes so that they don’t need to be relocated. For both simulations and real signals, the number of nodes had little effect in the ECGI solution, especially for torsos with more of 400 nodes, where the trend in the studied metrics was stabilized. This suggests that torso meshes built upon the restriction of including the electrodes as nodes in the torso mesh are reliable even for very low densities. Furthermore, we observed that irregular meshes presented better results in terms of metrics compared to regular meshes for finer geometries, likely because of a better shape preservation.

Additionally, we explored the effect of node distribution and density, considering that the remeshing affects the position of the nodes that corresponded to electrodes. This analysis is relevant in the context of building first a torso mesh and later assigning the nodes corresponding to recording electrodes in a second step. Under these constraints, the effect of torso density is no longer negligible, and CC can decrease down to 0.5 for meshes with 100 nodes in simulations when there are no further spatial uncertainties and noise is limited to 20 dB SNR. The impact of mesh density on real patient data, when different sources of uncertainty are present, is less relevant because correlations are lower than in the computer simulations even for torso meshes with 1,000 nodes. In either case, correlation coefficients are affected, and decrease from 0.87 ± 0.2 for 1,000 node meshes down to 0.42 ± 0.38.

The effect of the node density of the meshes of the torsos has not been widely studied. Nevertheless, an accurate torso geometry has been reported as necessary to obtain precise inverse electrograms (Messinger-Rapport and Rudy, 1990). Our study uses torso models obtained with photogrammetry, which presented realistic results but not as precise as those obtained with medical imaging techniques, which were reported to be very important for correct inverse results (Svehlikova et al., 2012). Previous studies addressed that as long as geometrical parameters are captured with local details, the significant impact on the inverse electrogram is minimal (Wang et al., 2010), which is in accordance with the presented results, especially with a larger number of nodes. Likewise, torso reshaping and remeshing with a different number of nodes affected the quality of the signals, with 400 nodes being the minimum necessary to obtain a reliable result. Torso reshaping and smoothing the geometry have been reported to produce less accurate results when computed inverse electrograms (Lenkova et al., 2012) were compared to real ones. Nevertheless, we demonstrated that a homogeneous distribution of the nodes improved the inverse solution for meshes of less than 1,000 nodes independently of the type of signal used (real or simulated) when the remeshing forced a relocation of the electrodes. Heterogenous distribution of the nodes improved the results compared to the homogeneous one for geometries of more than 1,000 nodes and torsos with the electrodes matching a node position. However, when number of nodes increased, the differences between the distribution of the nodes decrease, and we cannot ensure that homogeneous meshes are worse for higher number of nodes, most likely because electrode relocation is less relevant in homogenous torso meshes since the distance between the actual location of the electrode and its location in the relocated torso mesh is larger in heterogenous meshes than in their homogeneous counterparts.

The minimum number of electrodes for computing ECGI with AF signals needed has been studied previously, with 23 the minimum number for an accurate reconstruction, similar to a 12-lead ECG (Guillem et al., 2009). Although the number of electrodes remains critical for a proper inverse reconstruction, in this study, we used a reliable amount according to the literature. Notwithstanding, increasing the number may alleviate the misplacement effect and could be needed for a correct reconstruction of reliable torso meshes.

The position and displacement of the electrodes remain important, as shown in the results and described by van der Graaf et al., 2016. Nevertheless, some studies provided results that the optimal position for placing the electrodes is not unique, which matches the study (Lux et al., 1978). The possibility of a range of appropriate electrode positions allows the opportunity of having reliable ECGI reconstructions with different torso meshes and electrodes displacement as in the presented study. The remeshing influenced electrode location, but we could establish the maximum displacement tolerated of the electrodes as 2cm, the mean displacement of 400-nodes torsos, which is in accordance with what has been described *in vivo* studies previously (Cluitmans and Volders, 2017). This distance gives margin to consider as a good reconstruction of the location of the electrodes using photogrammetry. Furthermore, slightly displaced electrodes would not affect the results drastically. Despite that, results demonstrated that 400 nodes -or mean distance between nodes below 4 cm-is a good trade-off for torso geometry reconstruction; geometries with a higher number of nodes would alleviate electrode misplacement (Huiskamp and Van Oosterom, 1989).

### 4.1 Limitations

In this study, we compared real AF signals with ECGI reconstructed with a higher number of nodes in the mesh as a reference and with no intracardiac data. We considered a higher number of nodes models as a reference assuming that it will provide a better reconstruction. For this purpose, data from AF simulations were used, being the conclusions with simulations and real data in agreement.

For the used forward model, inner organs were not included. Our model may be simplistic, and for that reason, the observed results with relocated electrodes may be better in simulations than in patient analysis. Nevertheless, although the incorporation of inner organs has not shown a major impact on the shape of ECGI potentials (Ramanathan and Rudy, 2001), simulated body surface potentials are indeed affected by these torso inhomogeneities that we have not considered in the present study. Additionally, the lack of anisotropy of the forward model may influence our results because although it may not affect the ECGI resolution significantly, potential distributions that are more complex due to the anisotropy will complicate the resolution of the inverse model (Colli-Franzone et al., 1982; Hren et al., 1998; Potse et al., 2009). Furthermore, it should be noted that the presented results are not relevant to mesh-less solutions due to the influence of the BEM on the presented results. For simulations, the results for each torso geometry were compared with a reference ECGI of a 4000-nodes torso and not with the original electrogram due to the low similarity at the high-frequencies for the intrinsic smoothing of the ECGI. Nevertheless, this does not imply that we could define the effect of the quality of the mesh on the inverse solution. Finally, in the present study we have omitted the quantification of the impact of the epicardial mesh on the signals estimated by ECGI, which should be explored in future studies.

## 5 Conclusion

The present study shows that the effect of mesh density on ECGI signals has little effect when the original electrode position is respected, especially for geometries with more than 400 nodes. Nevertheless, if maintaining the original position of the electrode is not possible, a mesh of at least 400 nodes is recommended for solving the inverse problem of electrocardiography in the context of atrial fibrillation signals in order to achieve reliable results. Furthermore, a homogeneous distribution of the nodes showed to be convenient for computing the ECGI with a distance separation of nodes under 4 cm. A displacement of the nodes corresponding to the position of the electrodes higher than 2 cm should be avoided.

## Data Availability

The original contributions presented in the study are included in the article/supplementary material, further inquiries can be directed to the corresponding author.
